# Roux-en-Y Hepaticojejunostomy for Iatrogenic Bile Duct Injury (Strasberg E1): A Case Report and Review of the Surgical Technique

**DOI:** 10.7759/cureus.117594

**Published:** 2026-10-07

**Authors:** Martin Jesus Mendez Bacelis, Daniel Aguilar Rojas, Yunuén Maqueda Sánchez, Karla A Castillo Castro, Sergio Morales Acosta, Adolfo Peña Medina, Carlos Alberto Milán Lecou

**Affiliations:** 1 General Medicine, Universidad Autónoma del Estado de Quintana Roo, Cancún, MEX; 2 General Surgery, Cancun General Hospital "Jesus Kumate Rodriguez", Cancún, MEX; 3 General Practice, Anáhuac University, Mexico City, MEX; 4 General Practice, Faculty of Medicine, Universidad de Guadalajara Centro Universitario de Ciencias de la Salud (CUCS), Guadalajara, MEX; 5 General Surgery, Hospital General de Zona No.1 "Dr Carlos De La Peña", Saltillo, MEX

**Keywords:** bile duct injury repair, cholecystectomy complication, hepato-pancreato-biliary surgery, roux-en-y hepaticojejunostomy, strasberg e1

## Abstract

Iatrogenic bile duct injury (IBDI) is a severe complication of cholecystectomy that requires systematic management to avoid long-term morbidity and mortality. We report the case of a 47-year-old female who sustained a Strasberg E1 bile duct disruption following a cholecystectomy performed 15 days prior. Initial stabilization and biliary decompression were achieved via ultrasound/fluoroscopy-guided percutaneous transhepatic biliary drainage (PTBD). Following resolution of hyperbilirubinemia and targeted antibiotic therapy for multidrug-resistant biliary colonizers (*Escherichia coli *extended-spectrum beta-lactamase (ESBL) and *Enterococcus faecalis*), the patient underwent definitive open Roux-en-Y hepaticojejunostomy (Hepp-Couinaud technique) with a transanastomotic biliary catheter. Postoperative recovery was uneventful, demonstrating normalization of liver function and successful biliary drainage. This case underscores the value of a multidisciplinary step-up approach, adequate infection control, and adherence to surgical principles in achieving optimal outcomes in complex biliary injuries.

## Introduction

Iatrogenic bile duct injury (IBDI) remains one of the most serious complications following laparoscopic and open cholecystectomy, with an incidence ranging from 0.3% to 0.7% globally [1]. Factors contributing to IBDI include acute or chronic inflammation, severe anatomical distortion in Calot's triangle, aberrancies in biliary anatomy, and misidentification of the common bile duct or aberrant right hepatic ducts [2,3].

Unrecognized or improperly managed biliary injuries can quickly progress to biliary peritonitis, sepsis, secondary biliary cirrhosis, and recurrent cholangitis [4]. Classification systems provide standardized frameworks to define the level and extent of injury, guiding optimal timing and repair strategies [5]. The Strasberg classification categorizes injuries from types A to E based on ductal continuity and bile leak origins, with type E representing major ductal transections. Type E injuries directly correlate with the Bismuth classification, which stratifies disruptions according to their distance from the hepatic duct confluence: Bismuth type I (Strasberg E1) lesions occur more than 2 cm distal to the confluence with preserved confluence integrity, while types II through V represent progressively proximal injuries or segmental duct involvements. For major disruptions, Roux-en-Y hepaticojejunostomy (RYHJ) using the Hepp-Couinaud technique remains the gold-standard definitive surgical procedure [6]. This report details the clinical presentation, perioperative optimization, microbiologic management, and surgical repair of a Strasberg E1 bile duct injury in a 47-year-old female patient.

## Case presentation

A 47-year-old female with no prior chronic medical conditions presented to the emergency department with persistent right upper quadrant abdominal pain, fever, generalized jaundice, and bilious output from an abdominal drain. Fifteen days prior, she underwent a cholecystectomy at a regional hospital.

Upon clinical evaluation in the emergency department, vital signs demonstrated hemodynamic instability secondary to systemic inflammatory response syndrome (SIRS) (Table 1).

**Table 1:** Admission vital signs

Parameter	Patient Value	Reference Range	Units
Blood Pressure	100/78	90/60-120/80	mmHg
Heart Rate	108	60-100	beats/min
Respiratory Rate	20	12-20	breaths/min
Body Temperature	38	36.5-37.5	°C
Oxygen Saturation ($SpO_2$)	97 (Room Air)	95-100	%

Physical examination revealed generalized icteric sclerae and skin. Abdominal examination showed diffuse tenderness, most pronounced in the right hypochondrium with localized peritoneal signs (guarding and rebound tenderness), alongside an active surgical drain discharging 250 mL/24 hours of frank bilious fluid.

Laboratory evaluation on admission confirmed leukocytosis, marked systemic inflammation, and a severe cholestatic pattern with conjugated hyperbilirubinemia (Table 2).

**Table 2:** Admission laboratory findings

Parameter	Patient Value	Reference Range	Units
White Blood Cells (WBC)	16.8	4.5-11.0	10^3^/µL
Neutrophils	85.2	40.0-70.0	%
Hemoglobin	11.4	12.0-15.5	g/dL
Platelets	340	150-450	10^3^/µL
Total Bilirubin	8.45	0.20-1.20	mg/dL
Direct Bilirubin	6.10	0.00-0.30	mg/dL
Indirect Bilirubin	2.35	0.20-0.80	mg/dL
Aspartate Aminotransferase (AST)	142	10-40	U/L
Alanine Aminotransferase (ALT)	188	7-56	U/L
Alkaline Phosphatase (ALP)	485	44-147	U/L
Gamma-Glutamyl Transferase (GGT)	310	9-48	U/L
C-Reactive Protein (CRP)	128.5	0.0-5.0	mg/L

A contrast-enhanced abdominal computed tomography (CT) scan was performed, demonstrating a subhepatic fluid collection (biloma) in the gallbladder fossa, pericholedochal edema, and surgical clips at the porta hepatis with abrupt discontinuity of the extrahepatic biliary tree, suggestive of high-grade biliary injury (Figure 1). Definitive confirmation of complete common hepatic duct transection and Strasberg E1 classification was subsequently established intraoperatively.

**Figure 1:**
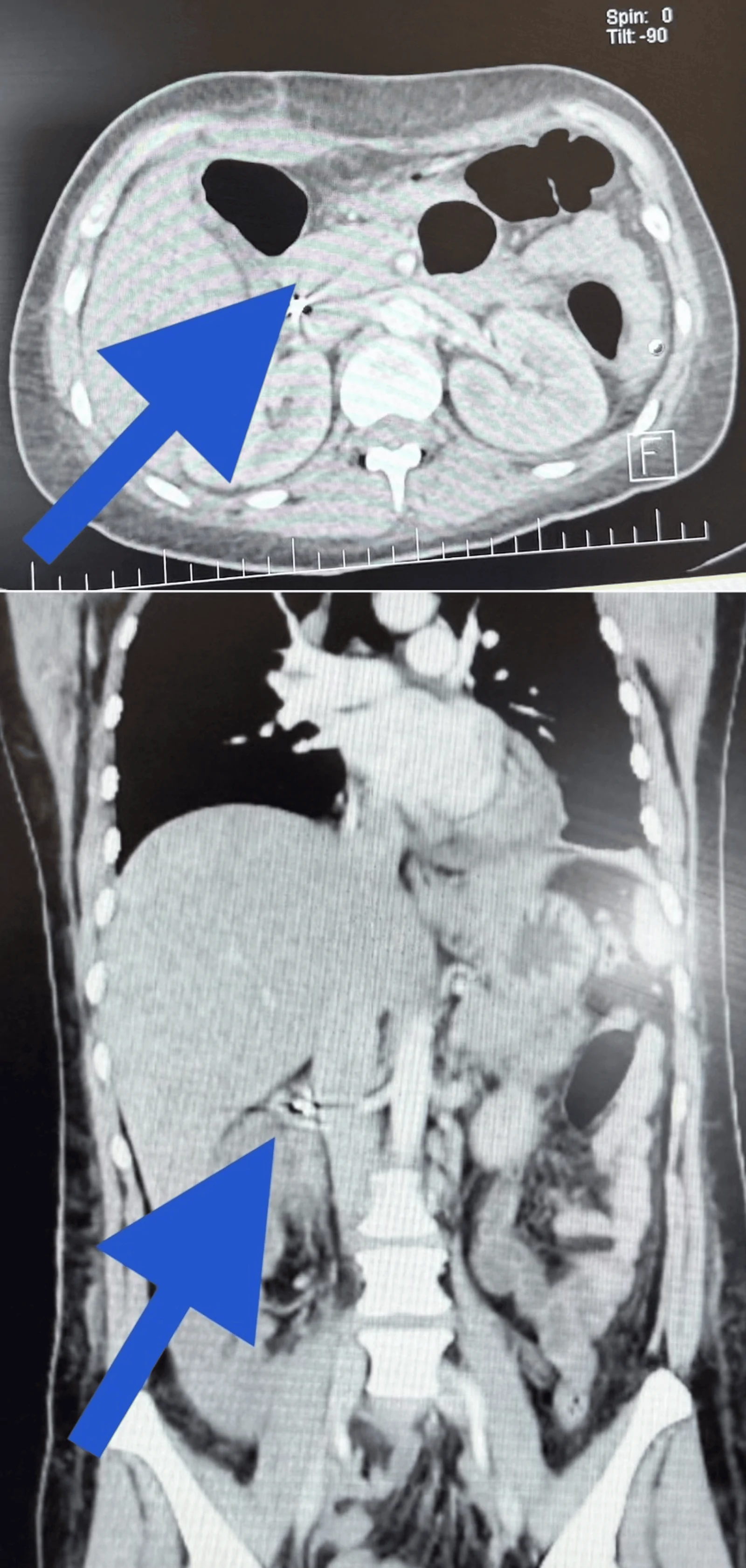
Abdominal computed tomography scan (A) Axial view showing a subhepatic fluid collection and surgical clips at the hilum. (B) Coronal view showing biliary disruption and proximal ductal dilation.

Initial management focused on hemodynamic stabilization, fluid resuscitation, broad-spectrum intravenous antibiotic coverage, and ultrasound/fluoroscopy-guided percutaneous transhepatic biliary drainage (PTBD) for biliary decompression and biloma drainage. Intraoperative bile cultures grew extended-spectrum beta-lactamase (ESBL)-producing *Escherichia coli* and *Enterococcus faecalis*, prompting targeted antibiotic therapy based on sensitivity profiles.

Once hyperbilirubinemia was resolved, sepsis was controlled, and coagulation parameters were normalized (Table 3), the patient was scheduled for definitive surgical reconstruction.

**Table 3:** Coagulation parameters and follow-up liver function tests ALT: alanine transaminase; AST: aspartate aminotransferase

Parameter	Admission Value	Pre-operative Value	Post-operative Day 30	Reference Range	Units
Prothrombin Time (PT)	14.8	12.1	11.8	11.0-13.5	sec
International Normalized Ratio (INR)	1.32	1.05	1.00	0.80-1.20	-
Activated Partial Thromboplastin Time	38.2	30.5	29.8	25.0-35.0	sec
Total Bilirubin	8.45	2.10	0.40	0.20-1.20	mg/dL
Direct Bilirubin	6.10	1.15	0.10	0.00-0.30	mg/dL
AST	142	48	22	10-40	U/L
ALT	188	52	37	7-56	U/L

Surgical technique

Exploratory laparotomy and adhesiolysis: Right subcostal incision, extensive adhesiolysis of the subhepatic and supramesocolic spaces, exposing the porta hepatis.

Identification of biliary stump and Hepp-Couinaud approach: Intraoperative exploration confirmed complete transection of the main hepatic duct >2 cm below the biliary confluence (Strasberg E1). Following the principles of the Hepp-Couinaud technique, the hilar plate was lowered at the base of segment IV to expose the extrahepatic course of the left hepatic duct. A longitudinal ductotomy measuring approximately 18 mm was performed along its anterior superior margin extending toward the confluence to ensure a wide, spatulated, and tension-free bilioenteric opening.

Roux loop preparation: The jejunum was transected 15-20 cm distal to the ligament of Treitz. A 60-cm retrocolic alimentary Roux limb was fashioned to ensure tension-free apposition.

Anastomosis construction: An end-to-side RYHJ was constructed under optical magnification. Surgical alternatives for bilioenteric reconstruction include interrupted and continuous suturing techniques; in this patient, a running (continuous) absorbable monofilament suture was chosen due to adequate ductal caliber and clear mucosal visualization, facilitating uniform circumferential tension distribution, reduced operative time, and a watertight seal while minimizing tissue trauma. A radiopaque transanastomotic silastic biliary catheter was placed across the anastomosis for temporary internal decompression and ductal calibration.

Reconstitution: Intestinal continuity was restored via a side-to-side jejunojejunostomy 60 cm distal to the biliary anastomosis. Closed-suction drains were placed adjacent to the reconstructed site.

The postoperative recovery was uneventful. Oral intake was resumed on Day 3, drains were removed on Day 6, and the patient was discharged with normal liver function and successful biliary drainage. At follow-up on postoperative Day 30, the patient remained asymptomatic with complete normalization of liver biochemistry (Table 3).

## Discussion

The key determinants of outcome in BDI are the timing of recognition, immediate control of biliary sepsis, and referral to a specialized hepato-pancreato-biliary (HPB) surgical team [7]. Immediate primary repair in the presence of active peritonitis or severe local tissue inflammation carries a high rate of anastomotic breakdown, stricture formation, and re-operation [8].

In this case of a Strasberg E1 injury (>2 cm from the primary ductal confluence), preoperative decompression via PTBD provided adequate biliary drainage, allowing local tissue inflammation to subside before definitive repair. Utilizing the Hepp-Couinaud technique allowed for a wide, low-tension, mucosa-to-mucosa anastomosis along the left hepatic duct.

The placement of a transanastomotic biliary catheter provided internal-external stenting, ensuring alignment and reducing the risk of immediate bile leakage or early anastomotic stricture [6,9]. Furthermore, targeted antimicrobial coverage based on bile cultures for *E. coli* ESBL and *E. faecalis* was critical to prevent postoperative infectious complications [4,10].

## Conclusions

IBDI during cholecystectomy remains a complex surgical challenge requiring timely diagnosis and specialized multidisciplinary management. In this patient with a Strasberg E1 lesion, early stabilization, preoperative biliary decompression, targeted antibiotic therapy, and definitive surgical reconstruction via RYHJ achieved favorable early postoperative recovery with complete normalization of liver function by postoperative Day 30. However, extended multidisciplinary follow-up over several years remains mandatory to monitor for late anastomotic strictures and recurrent biliary complications.
